# Crystal structures of human Fabs targeting the Bexsero meningococcal vaccine antigen NHBA

**DOI:** 10.1107/S2053230X17006021

**Published:** 2017-05-11

**Authors:** Martina Maritan, Roberta Cozzi, Paola Lo Surdo, Daniele Veggi, Matthew James Bottomley, Enrico Malito

**Affiliations:** a GSK Vaccines, Via Fiorentina 1, 53100 Siena, Italy

**Keywords:** fragment antigen binding, Fabs, monoclonal antibodies, mAbs, NHBA, *Neisseria*, vaccine, Bexsero

## Abstract

Neisserial heparin-binding antigen (NHBA) is a surface-exposed lipoprotein from *Neisseria meningitidis* and is a component of the meningococcus B vaccine Bexsero. As part of a study to characterize the three-dimensional structure of NHBA and the molecular basis of the human immune response to Bexsero, the crystal structures of two fragment antigen-binding domains (Fabs) isolated from human monoclonal antibodies targeting NHBA were determined. Through a high-resolution analysis of the organization and the amino-acid composition of the CDRs, these structures provide broad insights into the NHBA epitopes recognized by the human immune system. As expected, these Fabs also show remarkable structural conservation, as shown by a structural comparison of 15 structures of apo Fab 10C3 which were obtained from crystals grown in different crystallization conditions and were solved while searching for a complex with a bound NHBA fragment or epitope peptide. This study also provides indirect evidence for the intrinsically disordered nature of two N-terminal regions of NHBA.

## Abstract 2

The crystal structures and functional characterization of two anti-NHBA human Fabs provide insight into the recognition by human antibodies of one of the main components of the first vaccine against serogroup B *N. meningitidis* (Bexsero).

## 1. Introduction   

Serogroup B *Neisseria meningitidis* (MenB) is a Gram-negative encapsulated bacterium that can cause invasive meningococcal disease, which is characterized by severe infection and fatal sepsis (Rosenstein *et al.*, 2001 ▸). Vaccination is the most effective route to prevent meningococcal disease (Delany *et al.*, 2013 ▸), and the first recombinant vaccine against meningococcus B, known as 4CMenB or Bexsero, received regulatory approval by the European Medicines Agency in 2013 (European Medicines Agency, 2013 ▸). Bexsero is a multi-component vaccine that contains three surface-exposed recombinant proteins [factor H-binding protein (fHbp), neisserial heparin-binding antigen (NHBA) and neisserial adhesin A (NadA)] and outer membrane vesicles from a New Zealand strain (NZ OMV) (Giuliani *et al.*, 2006 ▸; Pizza *et al.*, 2000 ▸).

NHBA is a lipoprotein that is specific to *Neisseria* species, and its gene is ubiquitous in all meningococcal group B strains (Bambini *et al.*, 2009 ▸; Jacobsson *et al.*, 2006 ▸; Lucidarme *et al.*, 2010 ▸; Muzzi *et al.*, 2013 ▸). NHBA is able to generate protective immunity in animals and humans through the elicitation of bactericidal antibodies (Serruto *et al.*, 2010 ▸; Giuliani *et al.*, 2010 ▸; Welsch *et al.*, 2003 ▸). Gene-sequence analysis from genetically diverse group B strains revealed the existence of more than 600 NHBA variants, also termed peptides (Muzzi *et al.*, 2013 ▸; Bambini *et al.*, 2013 ▸). Examination of these gene sequences also revealed the presence of variable segments of NHBA at the level of the amino-acid sequence, with the highest sequence diversity clustered in the N-terminal region of the protein (approximately residues 1–240). In particular, sequence variability has been associated with an insertion of ∼60 amino acids (residues 115–177) that is present only in some meningococcal strains and that allows the classification of NHBA peptides into so-called ‘long’ and ‘short’ variants. The long NHBA peptide 2 (p2) variant is the most frequently expressed peptide found in molecular epidemiological studies in Europe, and it is the variant included in Bexsero (Vogel *et al.*, 2013 ▸). The amino-acid sequences of NHBA allow the identification of two independent domains (N-terminal and C-terminal domains) separated by a central polyarginine motif (Supplementary Fig. S1*a*). The three-dimensional structure of the highly conserved C-terminal domain has previously been determined by NMR (NHBA variant p20; residues 276–427, corresponding to residues 341–492 of variant p2), revealing a single eight-stranded antiparallel β-barrel immediately preceded by two β-strands in a hairpin conformation which, in the ensemble of NMR structures calculated, displayed multiple distinct locations with respect to the β-barrel (Esposito *et al.*, 2011 ▸). In contrast, the structure of the N-terminal domain remains unknown, and secondary-structure predictions suggest that it is highly flexible and contains intrinsically disordered regions (Esposito *et al.*, 2011 ▸; Supplementary Fig. S1*b*).

Intrinsically disordered proteins (IDPs) do not have a preferred three-dimensional structure, but rather exist in solution as an ensemble of interconverting conformers, which generally makes them difficult to crystallize (Mittag & Forman-Kay, 2007 ▸). Since IDPs might undergo transient binding-induced folding upon interaction with specific binding partners (Dyson & Wright, 2005 ▸), complex formation may promote their stabilization, as reported previously (Hurley *et al.*, 2007 ▸; Hanna *et al.*, 2008 ▸; Florek *et al.*, 2011 ▸). Monoclonal antibodies (mAbs) that bind IDPs can specifically recognize distinct conformers and thus act as surrogate binding partners (De Genst *et al.*, 2010 ▸; Sevcik *et al.*, 2009 ▸; Loris *et al.*, 2003 ▸). Additionally, fragments of monoclonal antibodies (Fabs) are well known as chaperones that can facilitate protein crystallization (Koide, 2009 ▸), and therefore have been used in this study to promote NHBA crystallization.

The progress that has been made in sequencing technologies in the last two decades has enabled the isolation and characterization of the antibody repertoires produced by antigen-specific B cells, thus allowing a better understanding of the molecular mechanisms of the immune responses to vaccine antigens to be gained (Rappuoli *et al.*, 2016 ▸). A recent study to profile the human B-cell repertoire in response to vaccination with the Bexsero antigens enabled the isolation of single plasmablasts from three patients immunized with MenB vaccine formulations. Thus, the sequencing of genes encoding Ig variable regions allowed the identification and characterization of 44 unique human monoclonal antibodies against fHbp, NHBA and NadA (Giuliani *et al.*, in preparation).

As part of the analyses of these Bexsero-specific antibodies and in an attempt to increase the structural characterization of NHBA, we selected two mAbs (named 12E1 and 10C3) which, according to preliminary epitope-mapping studies, bind to the NHBA N-terminal region between residues 1 and 300. We first cloned Fab fragments from these human mAbs, and then used these for binding, crystallization and co-crystallization studies with NHBA. In this work, we describe the preparation of the complexes between NHBA and Fabs 12E1 and 10C3, the characterization of their high-affinity interactions and the determination of the crystal structures of apo Fab 12E1 and Fab 10C3 at 2.7 and 1.5 Å resolution, respectively. Structural analyses provide insights into the NHBA epitopes recognized by these Fabs.

## 2. Materials and methods   

### 2.1. Protein expression and purification   

For the expression of the anti-NHBA human Fabs 12E1 and 10C3, gene fragments encoding the variable heavy chain (HC) and light chain (LC), as sequenced by Giuliani and coworkers (Giuliani *et al.*, in preparation), were codon-modified for optimal expression in *Homo sapiens*, synthesized by GeneArt (Life Technologies) and subcloned into pRS5a mammalian expression vector (Novartis AG). After resuspension in 50 µl nuclease-free water, the synthetic DNA strings were digested with the Eco31I restriction enzyme, purified and ligated into pRS5a expression vectors containing the Eco31I cloning site upstream of human IgG1 and Igκ/λ generic constant region sequences. The complete HC has a cleavable C-terminal Strep-tag II (Schmidt *et al.*, 1996 ▸). Expression of the Fabs was achieved by transient transfection exploiting Expi293 cells (Life Technologies) in suspension using Expi293 Expression Medium, according to the manufacturer’s protocol. Equal amounts (15 µg each per 30 ml of transfection volume) of vector DNA codifying FabH and FabL chains were used to transfect Expi293 cells. The cells were incubated at 37°C in a humidified atmosphere of 8% CO_2_ in air on an orbital shaker rotating at 125 rev min^−1^. The cell-culture supernatant was harvested 36 and 72 h post-transfection, clarified by centrifugation for 30 min at 4000 rev min^−1^ and concentrated using centrifugal concentration devices with a 10 kDa molecular-weight cutoff membrane (Millipore). Fabs 12E1 and 10C3 were both purified from the cell-culture supernatant by Strep-affinity chromatography, which was performed using a StrepTrap HP column (GE Healthcare) in 100 m*M* Tris, 150 m*M* NaCl, 1 m*M* EDTA pH 7.5, exploiting the highly selective double Strep-tag II at the C-terminus of the Fab heavy chain. The Strep-tag was removed using recombinant *Tobacco etch virus* (TEV) protease, which was prepared and purified in-house as described previously (van den Berg *et al.*, 2006 ▸). The Fabs were further purified by preparative size-exclusion chromatography on Superdex 200 resin in a 10/300 column (GE Healthcare) in buffer consisting of 20 m*M* Tris, 150 m*M* NaCl pH 8.

NHBA was produced in *Escherichia coli* BL21 (DE3)-T1^R^ cells (Invitrogen) using the EnPresso growth system (BioSilta) supplemented with 100 µg ml^−1^ ampicillin. The bacteria were grown at 30°C for 12 h and recombinant protein expression was induced by the addition of 1 m*M* isopropyl β-d-1-thiogalactopyranoside (IPTG) at 25°C for 24 h. The cells were harvested by centrifugation (6400*g*, 30 min, 4°C), resuspended in 50 m*M* NaH_2_PO_4_, 300 m*M* NaCl, 20 m*M* imidazole pH 7.5 and lysed by sonication (QSonica Q700). Cell lysates were clarified by centrifugation at 2800*g* for 30 min and the supernatant was filtered using a 0.22 µm membrane prior to protein purification. NHBA proteins were purified at room temperature (RT; 18–26°C) using an ÄKTApurifier 10 system (GE Healthcare) by nickel-affinity chromatography (5 ml HisTrap HP, GE Healthcare) followed by size-exclusion chromatography on Superdex 200 (16/60 column) equilibrated in 20 m*M* Tris–HCl, 150 m*M* NaCl pH 8.0. The homogeneity and the purity of the final samples were checked using SDS–PAGE 4–12% bis-tris gradient gels (Life Technologies) in 2-(*N*-morpholino)ethanesulfonic acid (MES) buffer.

The NHBA variants used in this study belonged to *N. meningitidis* strains MC58 (p3; UniProt Q9JQW0), NZ98/254 (p2, the vaccine variant; UniProt Q9JPH1) and 2996 (p20; UniProt Q9JPP1). Macromolecule-production information is summarized in Table 1 ▸.

### 2.2. Bioinformatics analyses   

The amino-acid sequence of NHBAp2 was analysed using bioinformatics tools for protein-fold and secondary-structure prediction. Namely, the following webservers were utilized: *Phyre*2 (Kelley *et al.*, 2015 ▸; Kelley & Sternberg, 2009 ▸), *SABLE* (Adamczak *et al.*, 2005 ▸), *PSIPRED* (Buchan *et al.*, 2013 ▸) and *JPred*4 (Drozdetskiy *et al.*, 2015 ▸).

### 2.3. Fab–NHBA binding studies   

The affinity of the binding of Fabs 12E1 and 10C3 to full-length NHBA proteins was determined by surface plasmon resonance (SPR), which was performed using a Biacore T200 instrument equilibrated at 25°C (GE Healthcare). Experiments were performed using a commercially available Human Fab Capture Kit (GE Healthcare) used to immobilize mAbs recognizing the κ and λ subtypes of Fab-fragment light chains by amine coupling on a carboxymethylated dextran sensor chip (CM5; GE Healthcare). A density level yielding ∼6000–7000 response units (RU) was prepared for immobilization on two flow cells of the CM5 chip. The immobilized anti-Fab mAbs were then used to capture 800–1100 RU of the tested human Fabs, injected at a concentration of 20 ng µl^−1^. The experimental SPR running buffer consisted of 10 m*M* HEPES, 150 m*M* NaCl, 3 m*M* EDTA, 0.05%(*v*/*v*) P20 surfactant pH 7.4 (HBS-EP). The equilibrium dissociation constant, *K*_d_, and the kinetic parameters were calculated by performing single-cycle kinetic (SCK) titration series of five consecutive injections of purified protein antigen diluted in HBS-EP at increasing concentrations (flow rate of 30 µl min^−1^, concentrations ranging from 6.25 to 100 n*M*) followed by a single final surface-regeneration step with buffer containing 10 m*M* glycine pH 2.1 (120 s; 10 µl min^−1^). Anti-human antibody-coated surfaces without captured Fab were used as a reference channel. A blank injection of buffer only was subtracted from each curve, and reference sensorgrams were subtracted from the experimental sensorgrams to yield curves representing specific binding. SPR data were analyzed using the *Biacore T200 Evaluation* software (GE Healthcare). Each sensorgram was fitted with a 1:1 Langmuir binding model, including a term to account for potential mass transfer, to obtain the individual kinetic constants *k*_on_ and *k*_off_. The individual values were then combined to derive the reported single averaged *K*_d_ values. The experiments were performed in duplicate.

### 2.4. Purification and crystallization of Fab–NHBA complexes   

Before crystallization experiments, Fab 12E1 or 10C3 was mixed with NHBA in a 1:1 molar ratio and the complex was purified by size-exclusion chromatography on Superdex 200 resin (10/300 column, GE Healthcare) equilibrated in 20 m*M* Tris–HCl, 150 m*M* NaCl pH 8.0. Purified complexes, as well as apo Fabs 10C3 and 12E1, were then used for crystallization screening using the commercial sparse-matrix crystallization screens Structure Screens 1 + 2, JCSG, ProPlex, SG1 and PACT *premier* from Molecular Dimensions and PEG/Ion from Hampton Research. Additionally, a purified sample of the 10C3–NHBAp2 complex was also used for *in situ* proteolysis experiments, in which the purified complex at a concentration of 30 mg ml^−1^ was treated with α-chymotrypsin (Jena Bioscience), which was added at a protein:protease ratio of 10000:1(*w*:*w*). The mixture was then immediately used to set up crystallization trials using the same crystallization screens as above. All crystallization experiments were performed at room temperature using a nanodroplet sitting-drop vapour-diffusion format. Equal volumes (200 nl) of protein sample and crystallization buffer were mixed with a Crystal Gryphon liquid dispenser (Art Robbins Instruments), and crystallization trays were imaged with a Rock Imager 182 automatic imaging system (Formulatrix).

Although the purification seemed to confirm the successful formation of the complexes with NHBA, only crystals of either apo Fab 12E1 or apo Fab 10C3 grew from these crystallization experiments. Specifically, apo Fab 12E1 crystals grew from a sample concentrated to 19 mg ml^−1^ as multiple and stacked plates from a condition consisting of 0.2 *M* potassium sodium tartrate, 0.1 *M* sodium citrate pH 5.6, 2 *M* ammonium sulfate (Table 2 ▸), while crystals of apo Fab 10C3 grew from a sample concentrated to 17 mg ml^−1^ in a number of different conditions (Supplementary Table S1). The condition that yielded the best-diffracting apo 10C3 crystals (1.5 Å resolution), and which were also used for the structure determination and refinement described below, consisted of 0.17 *M* ammonium sulfate, 15%(*v*/*v*) glycerol, 25.5%(*w*/*v*) PEG 4000 (Table 2 ▸).

### 2.5. Soaking experiments of NHBA epitope peptides into apo Fab crystals   

Because of the fragility of the Fab 12E1 crystals, soaking experiments were only performed using apo Fab 10C3 crystals. A peptide including residues 243–274 of NHBAp2 (KSEFEKLSDADKISNYKKDGKNDGKNDKFVGL) had previously been determined by hydrogen–deuterium exchange with mass spectrometry (HDX-MS) to be an epitope recognized by 10C3 (Giuliani *et al.*, in preparation). Additional considerations of the length of this fragment, and of the minimal sequence needed for binding, as obtained from multiple sequence alignments and from binding studies using different NHBA variants, led us to design a second shorter peptide containing residues 244–260 only (SEFEKLSDADKISNYKK). This was synthesized by JPT Peptide Technologies, and upon delivery in lyophilized form was first solubilized using 20 m*M* Tris–HCl, 150 m*M* NaCl pH 8.0 and then soaked in the mother liquor of apo Fab 10C3 crystals. Incubation times ranged from 5 min to 12 h, and the soaked drops were monitored under a microscope so that only crystals that did not suffer from these manipulations were subsequently frozen for X-ray data-collection experiments.

### 2.6. Data collection, processing, structure solution and refinement   

Before data collection, crystals of apo Fab 12E1 were cryoprotected using 10%(*w*/*v*) ethylene glycol, while those of apo Fab 10C3 were cryoprotected using either 20% glycerol or 20% ethylene glycol. The crystals were then flash-cooled in liquid nitrogen and diffraction data were collected on beamlines ID23-1 (12E1 crystals) or on beamlines BM30A and ID29 (10C3 crystals) at the European Synchrotron Radiation Facility (ESRF), Grenoble, France. All diffraction data were processed with *XDS* (Kabsch, 2010 ▸) and with programs from the *CCP*4 suite (Winn *et al.*, 2011 ▸). The structure of apo Fab 12E1 was solved using the automatic molecular-replacement (MR) pipeline *MoRDA* (Vagin & Lebedev, 2015 ▸), which automatically selected the coordinates of the human anti-human angiopoietin 2 Fab (PDB entry 4imk; Fenn *et al.*, 2013 ▸) as a search template. The structure of apo Fab 10C3 was also solved by MR using *Phaser* (McCoy *et al.*, 2007 ▸), with the coordinates of the human anti-HIV-1 clade A/E gp120 Fab N5-i5 (PDB entry 4h8w; Acharya *et al.*, 2014 ▸) as the input template search model. Manual model building of both structures was performed with *Coot* (Emsley *et al.*, 2010 ▸), refinement was performed with *PHENIX* (Adams *et al.*, 2010 ▸) and *BUSTER* (Bricogne *et al.*, 2016 ▸), and the quality of the final refined models was assessed using *MolProbity* (Chen *et al.*, 2010 ▸). All figures were generated using *PyMOL* (http://www.pymol.org). Data-collection and processing statistics and structure-refinement statistics are reported in Tables 3 ▸ and 4 ▸, respectively.

## 3. Results and discussion   

**Figure 1:**
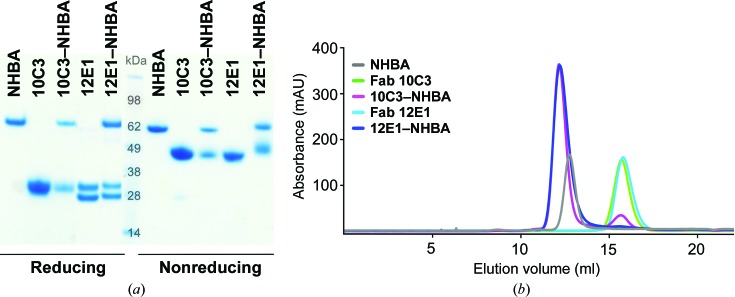
Formation and characterization of Fab–NHBA complexes. (*a*) SDS–PAGE analysis under reducing (left) and nonreducing (right) conditions of purified NHBAp2 (lane 1), Fab 10C3 (lane 2), the 10C3–NHBA complex (lane 3), Fab 12E1 (lane 4) and the 12E1–NHBA complex (lane 5). (*b*) Size-exclusion chromatography elution profiles of NHBAp2 (grey), Fab 10C3 (green), the 10C3–NHBA complex (magenta), Fab 12E1 (cyan) and the 12E1–NHBA complex (blue). Each chromatogram refers to an independent run.

Recombinant Fabs 12E1 and 10C3 were expressed by transient transfection of HEK-293 cells, and SDS–PAGE analyses of the purified Fabs confirmed their homogeneity, purity and expected homodimeric assembly (Fig. 1 ▸ *a*). After incubating Fab 10C3 or 12E1 with the purified vaccine variant NHBAp2, and after running these complexes through a size-exclusion chromatography column, SDS–PAGE analyses of the eluted fractions and of the chromatographic elution profiles (Figs. 1 ▸ *a* and 1 ▸ *b*) suggested that both complexes were formed. The binding of Fabs 12E1 and 10C3 to NHBAp2 was also studied by SPR, revealing equilibrium dissociation constants (*K*_d_) of 0.33 and 5.5 n*M* (Supplementary Table S2 and Supplementary Fig. S2), respectively. The binding affinities were also measured for NHBA sequence variants p3 (long variant) and p20 (short variant), showing that Fabs 12E1 and 10C3 recognize all variants tested with high binding affinity, except for NHBAp20, for which no binding by Fab 10C3 was detected. This binding specificity is presumably owing to sequence differences in the putative epitope region of NHBAp2 (243-KSEFE**K**L**SDADK**I**SN**YKKDG-262) with respect to that of NHBAp20 (180-KSEFE**N**L**NESER**I**EK**YKKDG-199). Various strategies were employed in order to determine the structures of Fab–NHBA complexes. Difficulties in obtaining crystals of Fab–NHBA complexes, likely owing to the lack of stable structured elements in the N-terminus of NHBA (Supplementary Fig. S1), and the simultaneous availability of apo Fab crystals, prompted us to use the latter for soaking experiments. Also, in an attempt to free NHBA from poorly structured or flexible regions lying outside the epitope and thus to facilitate its crystallization, we explored the *in situ* proteolysis approach (Dong *et al.*, 2007 ▸). From these numerous attempts, only crystals and structures of the apo Fabs were obtained, analyses of which now allow insight into NHBA binding epitopes to be indirectly gained.

### 3.1. Crystal structure of Fab 12E1   

**Figure 2:**
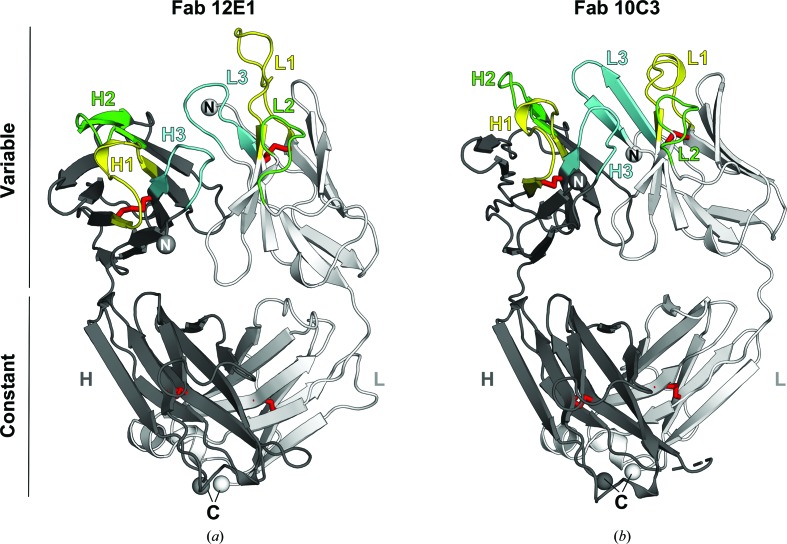
Overall fold of the apo Fab 12E1 and Fab 10C3 structures. The structures of Fab 12E1 (*a*) and Fab 10C3 (*b*) are depicted as cartoons, with the heavy (H) and light (L) chains coloured dark and light grey, respectively. CDR-L1 and CDR-H1 are coloured yellow, CDR-L2 and CDR-H2 green, and CDR-L3 and CDR-H3 cyan. Disulfide bonds are depicted by red sticks in each structure.

Crystals of apo Fab 12E1 diffracted to 2.7 Å resolution, belonged to space group *P*2_1_2_1_2 and contained a single 12E1 molecule in the asymmetric unit (Matthews coefficient of 2.66 Å^3^ Da^−1^, solvent content of 53.8%; Matthews, 1968 ▸). Full manual model building and refinement of the 12E1 structure yielded final *R*_work_ and *R*_free_ values of 18.0 and 26.3%, respectively (Table 4 ▸). Excellent and continuous electron-density maps allowed modelling of the Fab 12E1 molecule including residues Gln1–Lys216 for the heavy (H) chain and Glu1–Arg216 for the light (L) chain, while the final C-terminal residues of the H chain (residues Ser217–Gln228, including the TEV cleavage site) and three residues of the L chain (Gly217–Cys219) could not be modelled owing to a lack of electron density. The overall architecture and fold of the Fab 12E1 structure is consistent with the canonical β-sandwich immunoglobulin fold composed of two chains (H and L) and four domains (variable light, VL; constant light, CL; variable heavy, VH; constant heavy 1, CH1), with four pairs of intra-domain disulfide bridges clearly observed in the electron-density maps that link residues Cys22 and Cys96 in the VH domain, Cys142 and Cys198 in the CH1 domain, Cys23–Cys93 in the VL domain and Cys139–Cys199 in the CL domain (Fig. 2 ▸ *a*).

### 3.2. Crystal structure of Fab 10C3   

Crystals of apo 10C3 grew under a variety of conditions after 1–7 d of incubation [group (1) in Supplementary Table S1]. These crystals were used for soaking experiments, which were performed using the best-looking crystals and a 17-residue NHBA-derived peptide consisting of residues 244-SEFEKLSDADKISNYKK-260. Crystals were also obtained from *in situ* proteolysis experiments on the 10C3–NHBAp2 complex, and these grew after ∼60 d in several conditions [group (2) in Supplementary Table S1].

X-ray diffraction experiments using the Fab 10C3-containing crystals resulted in 15 data sets with resolutions of between 1.5 and 2.2 Å (Supplementary Table S1). An initial data set (data set 7 in Supplementary Table S1) at 1.7 Å resolution was selected for structure determination by MR in *Phaser* (McCoy *et al.*, 2007 ▸), and the full refined structure was then used as a template to solve eight structures (data sets 8–15) using data collected either from the peptide-soaked 10C3 crystals or from those obtained by *in situ* proteolysis. Disappointingly, none of the electron-density maps calculated from these data sets revealed the presence of bound NHBA or fragments thereof. In the case of the soaked crystals, the absence of density for a putative bound peptide might suggest a suboptimal conformation or/and design of the synthesized peptide owing to a lack of more precise information on the exact residues required for binding. Also, the physicochemical properties of this designed and synthesized peptide, such as the relative abundance of charged residues as well as its overall relatively large size, might have affected its ability to diffuse through the apo 10C3 crystal channels during soaking. Instead, for crystals obtained by the *in situ* proteolysis experiments, the slow growth of these crystals (∼60 d) suggests that extensive proteolysis might have taken place, resulting in the dissociation of NHBA and thus the crystallization of apo Fab 10C3 only.

Among the 15 apo 10C3 structures determined in this study, the highest resolution data set (1.5 Å) was selected for full refinement and validation and for PDB deposition. These crystals belonged to space group *P*2_1_2_1_2_1_, with a calculated Matthews coefficient of 2.33 Å^3^ Da^−1^ (solvent content 47.3%) and one 10C3 molecule occupying the asymmetric unit. The final coordinates of apo 10C3 were refined to final *R*_work_ and *R*_free_ values of 18.0 and 21.1%, respectively (Table 4 ▸). Excellent electron densities throughout most of the Fab chains allowed model building of residues Met2–Pro215 of the H chain and Ser2–Pro212 of the L chain, except for H-chain residues belonging to loop Ser130–Ser134 and to the terminal TEV cleavage site of the H chain (Lys216–Gln228). Clear electron-density maps could be observed for disulfide bonds linking residues Cys137–Cys197 of the CL domain, Cys142–Cys198 of the CH1 domain, Cys22–Cys90 of the VL domain and Cys22–Cys96 of the VH domain (Fig. 2 ▸ *b*).

**Figure 3:**
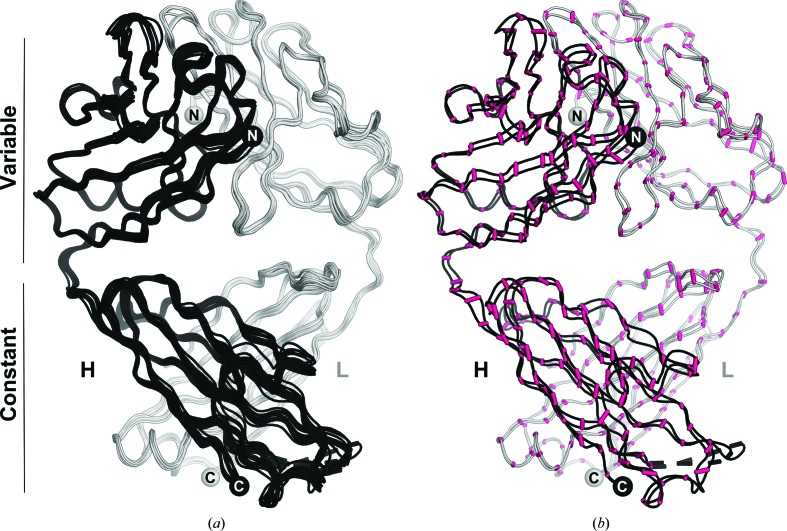
Structural comparisons of apo 10C3 structures. (*a*) All 15 10C3 structures solved in this work are shown as ribbons after superposition, and are coloured black and white for the heavy (H) and light (L) chains, respectively. (*b*) The two most divergent apo 10C3 structures are depicted superposed as ribbons (structures 6 and 15; see Supplementary Table S1) and coloured as in (*a*). The regions of maximum divergence between C^α^ atoms of the two structures are shown as magenta sticks.

The 15 refined crystal structures of the same apo Fab 10C3 (Supplementary Table S1 were compared by structural superposition to detect whether any structural differences existed both in their overall and local fold. The apo 10C3 structure at 1.5 Å resolution was thus used as reference coordinates for structural comparisons among all 10C3 structures, with superpositions performed by aligning the common C^α^ atoms with the secondary-structure matching (*SSM*) algorithm in *Coot* (Emsley *et al.*, 2010 ▸; Figs. 3 ▸ *a* and 3 ▸ *b*). The maximum r.m.s. deviations were observed for data sets 2 and 6, which diverged from the reference model with r.m.s.d. values of 1.01 and 0.97 Å, respectively, while the most superimposable structure (data set 7) had an r.m.s.d value of 0.23 Å (Supplementary Table S1). No clear correlation between the degree of structure similarity and the crystallization condition was identified. In conclusion, these analyses confirmed that, as expected, the overall fold of Fab 10C3 is conserved, an observation that agrees with the intrinsic and general structural stability of Fabs (Al-Lazikani *et al.*, 1997 ▸).

In summary, the structures of apo Fab 10C3 are highly isomorphous, although they were obtained from crystals obtained under different crystallization conditions, which include pH values ranging from 4.2 to 6.5 (Supplementary Table S1). Although several proteins undergo pH-induced conformational changes, this striking structural reproducibility has been reported previously for other Fabs (Skrabana *et al.*, 2012 ▸).

### 3.3. Structural analyses of Fab 12E1 and Fab 10C3 CDRs and putative paratopes   

**Figure 4:**
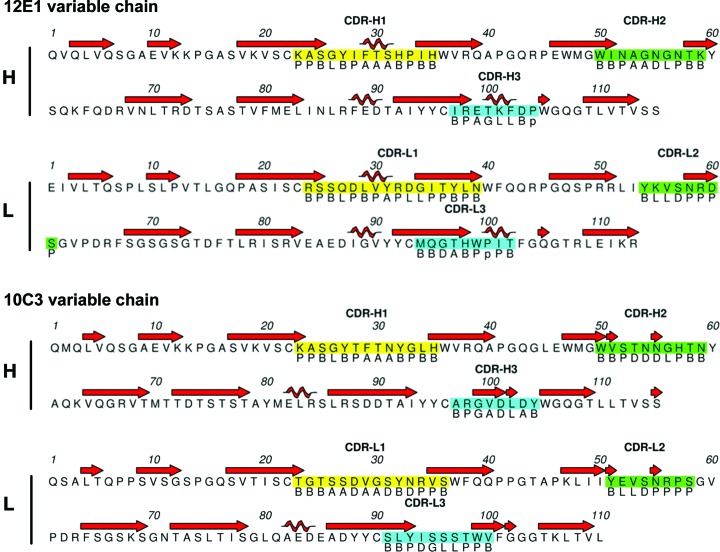
Sequences and structural annotations of the Fab 12E1 and Fab 10C3 CDRs. The sequences of Fab 12E1 (top) and Fab 10C3 (bottom) are shown with secondary-structure annotation at the top. CDR residues are highlighted in yellow (CDR-H1 and CDR-L1), green (CDR-H2 and CDR-L2), and cyan (CDR-H3 and CDR-L3). CDR conformations and secondary-structure elements are shown below and above the sequence, respectively. Regions of the Ramachandran plot that define CDR clusters by conformation are annotated as follows: B for β-sheet region, P for polyproline II, A for α-helix, D for δ region (near α-helix but with more negative values of φ), L for left-handed helix and G for γ region (φ > 0° excluding the L and B regions). Lower-case letters in the loop conformations indicate *cis* residues.

**Figure 5:**
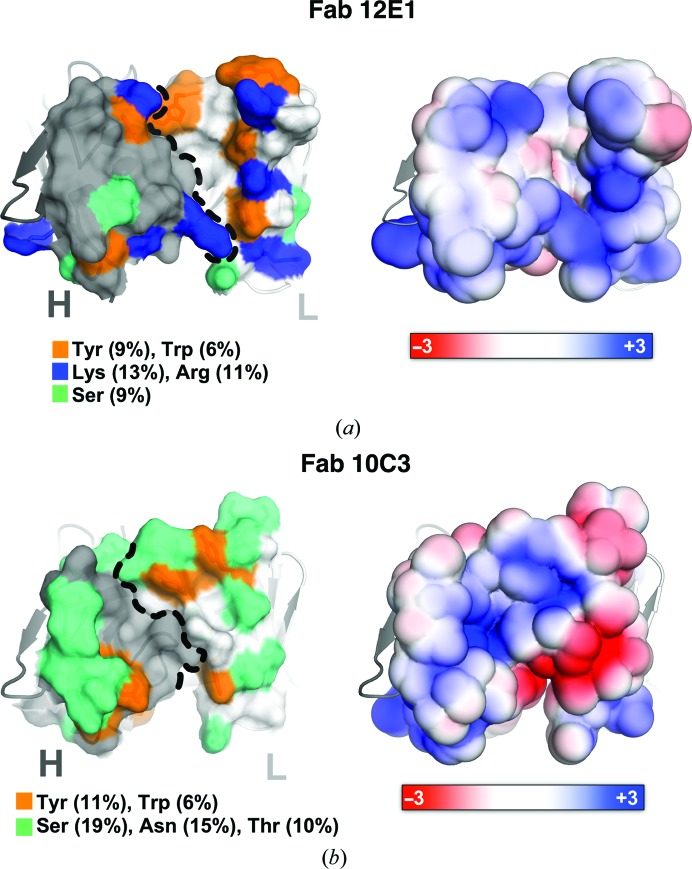
Fab 12E1 and Fab 10C3 CDRs. Top views of the Fab 12E1 (*a*) and Fab 10C3 (*b*) CDR regions, coloured according either to the most represented residues (left) or to the electrostatic potential distribution (right). Orange patches indicate aromatic residues (Trp and Tyr), blue patches indicate positively charged residues (Lys and Arg) and green patches indicate polar uncharged residues (Ser, Thr and Asn). Dotted black lines separate the Fab heavy and light chains. The electrostatic potential distribution was calculated with *APBS* (Lerner & Carlson, 2006 ▸), where red and blue surfaces show negative and positive charges contoured in the range from −3*k*_B_ *T* e^−1^ (red) to +3*k*_B_ *T* e^−1^ (blue), while white surfaces indicate neutral potential.

Although we were not able to obtain structures of Fab–NHBA complexes that could reveal the exact epitopes involved in immune recognition, only the structures of unbound or apo Fabs, we sought to utilize these structures in combination with other data in order to gain insight into the nature of their cognate epitopes. For this, we first performed analyses and annotations of the complementarity-determining regions (CDRs) of 12E1 and 10C3 and their respective loop conformations, using a recently introduced structure-based definition and nomenclature (North *et al.*, 2011 ▸; Figs. 4 ▸ *a* and 4 ▸ *b*; Supplementary Tables S3*a* and S3*b*). We then analysed the amino-acid compositions of the putative paratopes of the Fabs and those of the peptide epitopes previously determined by peptide scanning (PepScan) and HDX-MS to be recognized by 12E1 and 10C3 (Giuliani *et al.*, in preparation). According to these definitions, the CDR regions of Fabs 12E1 and 10C3 have calculated accessible surface areas (ASAs) of ∼3850 and ∼3600 Å^2^, respectively, as calculated with *PISA* (Krissinel & Henrick, 2007 ▸). Among the residues that are surface-exposed on the 12E1 CDRs, Lys and Arg are the most abundant, followed by Ser and Tyr (Fig. 5 ▸ *a* and Supplementary Table S4*a*). Interestingly, the enrichment of Fab paratopes with aromatic and Ser residues is in agreement with previous studies on the composition of antibody paratopes (Ramaraj *et al.*, 2012 ▸; Mian *et al.*, 1991 ▸; Kringelum *et al.*, 2013 ▸; Ofran *et al.*, 2008 ▸; Yu *et al.*, 2012 ▸). In more detail, the location of Ser on the surface of the Fab 12E1 CDRs appears to be mostly peripheral, while Tyr and Trp are more equally distributed on the top of the CDRs (Fig. 5 ▸ *a*). A more noticeable feature of the 12E1 structure is the presence of a high number of positively charged residues in the proximity of the putative paratope, mainly Arg and Lys (Fig. 5 ▸ *a*). This feature is not common among other Fabs, as long-chain hydrophilic residues are not frequently found in antibody paratopes (Peng *et al.*, 2014 ▸), and it suggests a possible role in the recognition of NHBA. Specifically, the presence of these positively charged patches in the paratope of 12E1 allows us to speculate on an apparent charge complementarity with the overall acidic nature of the linear epitope previously mapped on several NHBA variants (p1, p2, p3, p5, p18, p20, p21 and p29) consisting of residues 73-AAVSEENTGN-82 (Giuliani *et al.*, in preparation).

The CDRs of Fab 10C3 mostly consist of polar uncharged residues such as Asn, Ser and Thr (Fig. 5 ▸ *b* and Supplementary Table S3*b*). These residues are clustered in the loop regions of CDR-H1, CDR-H2, CDR-L1 and CDR-L3 and contribute, together with several Tyr residues, to create a rim around a central positively charged cavity at the interface between the H and L chains (Fig. 5 ▸ *b*). In addition, Asp101 and Asp103 of CDR-H3, and Glu52 of CDR-L2, contribute to the formation of a negatively charged lateral surface patch (Fig. 5 ▸ *b*). In an attempt to speculate on the binding of 10C3 to NHBA, the paratope composition analysed and described above can be related to the physicochemical properties of a previously identified putative epitope of 10C3 (peptide 243–274, consisting of KSEFEKLSDADKISNYKKDGKNDGKND­KFVGL; Giuliani *et al.*, in preparation). This peptide is particularly rich in charged residues, especially Lys and Asp, which might complement the exposed charged patches observed on the surface of the putative 10C3 paratope (Fig. 5 ▸ *b*). This suggests that electrostatic interactions might play a predominant role in recognition of NHBA by Fab 10C3, as also observed for Fab 12E1. Interestingly, this type of protein–protein interaction has been previously described as characteristic of antibody recognition of IDPs (Wong *et al.*, 2013 ▸; Peng *et al.*, 2014 ▸). Moreover, the lack of recognition of 10C3 by NHBAp20 might be owing to unfavourable electrostatic interactions, as the slight sequence differences between NHBAp2 (243-KSEFE**K**L**SDADK**I**SN**YKKDG-262) and NHBAp20 (180-KSEFE**N**L**NESER**I**EK**YKKDG-199) in the putative epitope region might result in a different electrostatic potential distribution on the antigen surface.

## 4. Conclusions   

In this work, we have studied the binding and determined the structures of two antigen-specific Fabs derived from human monoclonal antibodies raised against NHBA, one of the components of the meningococcal B vaccine Bexsero. To our knowledge, the structures reported here are the first crystal structures of anti-NHBA Fabs. In addition to providing the first, although indirect, evidence that the recognition of the N-terminal region of NHBA by the human immune system might take place according to the protein–protein interaction principles of IDPs, these structures also contribute to populate data sets required for training computational methods aimed at antibody modelling and B-cell epitope predictions.

## Supplementary Material

PDB reference: human Fab fragment 10C3, 5n4j

PDB reference: human Fab fragment 12E1, 5n4g

Supplementary tables.. DOI: 10.1107/S2053230X17006021/rl5137sup1.pdf

## Figures and Tables

**Table 1:** Macromolecule-production information

Expression vector	pRS5a
Expression host	Human embryonic kidney cells (Expi293F)
Complete amino-acid sequence of 10C3†	
Light chain	QSALTQPPSVSGSPGQSVTISCTGTSSDVGSYNRVSWFQQPPGTAPKLIIYEVSNRPSGVPDRFSGSKSGNTASLTISGLQAEDEADYYCSLYISSSTWVFGGGTKLTVLGQPKAAPSVTLFPPSSEELQANKATLVCLISDFYPGAVTVAWKADSSPVKAGVETTTPSKQSNNKYAASSYLSLTPEQWKSHRSYSCQVTHEGSTVEKTVAPTECS
Heavy chain	QMQLVQSGAEVKKPGASVKVSCKASGYTFTNYGLHWVRQAPGQGLEWMGWVSTNNGHTNYAQKVQGRVTMTTDTSTSTAYMELRSLRSDDTAIYYCARGVDLDYWGQGTLLTVSSASTKGPSVFPLAPSSKSTSGGTAALGCLVKDYFPEPVTVSWNSGALTSGVHTFPAVLQSSGLYSLSSVVTVPSSSLGTQTYICNVNHKPSNTKVDKRVEPKSCDKGSENLYFQGSWSHPQFEKGGGSGGGSGGGSWSHPQFEK
Complete amino-acid sequence of 12E1†	
Light chain	EIVLTQSPLSLPVTLGQPASISCRSSQDLVYRDGITYLNWFQQRPGQSPRRLIYKVSNRDSGVPDRFSGSGSGTDFTLRISRVEAEDIGVYYCMQGTHWPITFGQGTRLEIKRTVAAPSVFIFPPSDEQLKSGTASVVCLLNNFYPREAKVQWKVDNALQSGNSQESVTEQDSKDSTYSLSSTLTLSKADYEKHKVYACEVTHQGLSSPVTKSFNRGEC
Heavy chain	QVQLVQSGAEVKKPGASVKVSCKASGYIFTSHPIHWVRQAPGQRPEWMGWINAGNGNTKYSQKFQDRVNLTRDTSASTVFMELINLRFEDTAIYYCIRETKFDPWGQGTLVTVSSASTKGPSVFPLAPSSKSTSGGTAALGCLVKDYFPEPVTVSWNSGALTSGVHTFPAVLQSSGLYSLSSVVTVPSSSLGTQTYICNVNHKPSNTKVDKRVEPKSCDKGSENLYFQGSWSHPQFEKGGSGGGSGGGSWSHPQFEK

† The complete sequences of the recombinant Fab proteins produced are shown. Underlined residues indicate the residues that were removed upon cleavage by TEV protease (prior to crystallization screening and other experiments), including the double Strep-tag.

**Table 2:** Crystallization

	Fab 12E1	Fab 10C3
Method	Sitting-drop vapour diffusion	Sitting-drop vapour diffusion
Plate type	96-well low-profile Intelli-Plates (Art Robbins)	96-well low-profile Intelli-Plates (Art Robbins)
Temperature (K)	293.15	293.15
Volume and ratio of drop	1:1 ratio protein:reservoir (200 nl)	1:1 ratio protein:reservoir (200 nl)
Volume of reservoir (µl)	70	70
Buffer composition of protein solution	150 m*M* NaCl, 20 m*M* Tris–HCl pH 8	150 m*M* NaCl, 20 m*M* Tris–HCl pH 8
Protein concentration (mg ml^−1^)	19	17
Composition of reservoir solution	0.2 *M* potassium sodium tartrate, 0.1 *M* sodium citrate pH 5.6, 2 *M* ammonium sulfate	0.17 *M* ammonium sulfate, 15%(*v*/*v*) glycerol, 25.5%(*w*/*v*) PEG 4000

**Table 3:** Data collection and processing Values in parentheses are for the outer shell.

	Fab 12E1	Fab 10C3
Diffraction source	Beamline ID23-1, ESRF	Beamline BM30A, ESRF
Wavelength (Å)	0.97932	0.979788
Temperature (K)	100	100
Detector	PILATUS 6M-F	ADSC Quantum 315r
Crystal-to-detector distance (mm)	475.13	204.435
Rotation range per image (°)	0.15	0.5
Total rotation range (°)	128.1	180
Exposure time per image (s)	0.041	3
Space group	*P*2_1_2_1_2	*P*2_1_2_1_2_1_
*a*, *b*, *c* (Å)	64.8, 82.1, 100.3	69.9, 79.8, 82.5
α, β, γ (°)	90, 90, 90	90, 90, 90
Mosaicity (°)	0.28	0.184
Resolution range (Å)	45.41–2.75 (2.85–2.75)	44.36–1.50 (1.55–1.50)
Total No. of reflections	65074 (6325)	264152 (40399)
No. of unique reflections	14236 (1360)	73404 (11580)
Completeness (%)	98.6 (97.5)	99.0 (97.7)
Multiplicity	4.6 (4.7)	3.6 (6.3)
〈*I*/σ(*I*)〉†	9.9 (1.6)	14.88 (1.35)
*R*_meas_	0.151 (1.009)	0.05934 (1.167)
Overall *B* factor from Wilson plot‡ (Å^2^)	53.12	18.09

† 〈*I*/σ(*I*)〉 in the outer shells is <2.0 between 2.80 and 2.90 Å resolution for the Fab 12E1 data set and between 1.50 and 1.59 Å resolution for the Fab 10C3 data set.

‡ No anomalies were observed in the Wilson plot.

**Table 4:** Structure refinement Values in parentheses are for the outer shell.

	Fab 12E1	Fab 10C3
Resolution range (Å)	23.99–2.75 (2.85–2.75)	32.19–1.50 (1.55–1.50)
Completeness (%)	98.6 (97.5)	99.0 (97.7)
No. of reflections, working set	14204	73375
No. of reflections, test set	717	3668
Final *R*_work_	0.1808 (0.2197)	0.1788 (0.3057)
Final *R*_free_	0.2638 (0.3366)	0.2114 (0.3300)
No. of non-H atoms		
Protein	3337	3156
Ligand	26	—
Water	73	701
Total	3436	3857
R.m.s. deviations		
Bonds (Å)	0.0014	0.010
Angles (°)	1.96	1.27
Average *B* factor (Å^2^)		
Overall	56.44	23.88
Protein	56.49	21.24
Ligand	70.63	—
Water	48.34	35.80
Ramachandran plot		
Most favoured (%)	93.4	98
Allowed (%)	5.6	1.9
PDB entry	5n4g	5n4j
