# Supplementary material for: Crystal structures of human Fabs targeting the Bexsero meningococcal vaccine antigen NHBA
Source: Acta Crystallogr F Struct Biol Commun. 2017 May 11;73(Pt 6):305–14. doi: 10.1107/S2053230X17006021 (PMC5458386; doi:10.1107/S2053230X17006021)
Supplement: Supplementary file 1 [file f-73-00305-sup1.pdf]

STRUCTURAL BIOLOGY
COMMUNICATIONS

Volume 73 (2017)

Supporting information for article:

Crystal structures of human Fabs targeting the Bexsero™ meningococcal vaccine antigen NHBA

Martina Maritan, Roberta Cozzi, Paola Lo Surdo, Daniele Veggi, Matthew James Bottomley and Enrico Malito

Table S1 Apo-Fab 10C3 structures solved in this work

.Column I: starting material used for the crystallization experiment; Column II: identification number of the dataset; Column III: crystallization conditions; Column IV: resolution of each dataset; Column V and VI: cell dimensions and space group (SG); column VII: rmsd values obtained by SSM of each dataset superposed by SSM onto the reference structure from dataset #15.

starting crystallization material	# dataset	Mother liquor composition	Resolution	cell dimension (a b c)	SG	SSM r.m.s.d. (Å)
(1) Crystals of apo-Fab 10C3	1	0.2 M $LiSO_4$,0.1 M BIS-TRIS 5.5 pH, 25 %w/v PEG 3350	1.9Å	69.30 76.58 82.14	$P\ 2_1\ 2_1\ 2_1$	0.33
	2	0.1 M KSCN, 30 %w/v PEG MME 2000	1.8Å	69.67 76.78 82.29	$P\ 2_1\ 2_1\ 2_1$	1.01
	3	0.1 M KSCN, 30 %w/v PEG MME 2000	1.9Å	69.72 77.69 82.49	$P\ 2_1\ 2_1\ 2_1$	0.76
	4	0.2 M NH_4 Acet, 0.1 M BIS-TRIS 5.5 pH, 25 %w/v PEG 3350	1.9Å	69.65 77.91 82.35	$P\ 2_1\ 2_1\ 2_1$	0.67
	5	0.2 M $(NH4)_2$H Cit, 20 %w/v PEG 3350	1.8Å	69.65 77.31 82.05	$P\ 2_1\ 2_1\ 2_1$	0.88
	6	37.5 %w/v M1K3350, 0.1 M MB1 6.5 pH, 10 % MAA	2.2 Å	69.61 74.24 82.41	$P\ 2_1\ 2_1\ 2_1$	0.97
	7	0.2 M NH_4 Acet, 0.1 M Na_3 Cit 5.6 pH, 30 %w/v PEG 4000	1.7 Å	69.74 78.69 82.89	$P\ 2_1\ 2_1\ 2_1$	0.23
(2) Crystals of complexed Fab 10C3	8	0.2 M Ammonium sulfate 0.1 M Sodium acetate pH 4.6, 25 % w/v PEG 4000	1.89 Å	69.17 76.8 82.53	$P\ 2_1\ 2_1\ 2_1$	0.56
	9	0.2 M Ammonium sulfate 30 % w/v PEG 4000	1.53 Å	67.93 75.54 81.87	$P\ 2_1\ 2_1\ 2_1$	0.44
	10	0.2 M Magnesium chloride hexahydrate 0.1 M MES pH6.0 20 % w/v PEG 6000	1.69 Å	69.33 76.81 82.28	$P\ 2_1\ 2_1\ 2_1$	0.76
	11	0.2 M Magnesium chloride hexahydrate 0.1 M MES pH6.0 20 % w/v PEG 6000	1.28 Å	69.49 78.11 82.86	$P\ 2_1\ 2_1\ 2_1$	0.36
	12	0.2 M Calcium chloride dihydrate 0.1 M MES pH6.0 20 % w/v PEG 6000	1.44 Å	76.77 82.04 68.80	$P\ 2_1\ 2_1\ 2_1$	0.58
(3) Crystals of soaked Fab 10C3	13	0.2 M NaCl, 0.1 M Na Phos Cit 4.2 pH , 20 %w/v PEG 8000	2.2Å	70.19 78.15 83.86	$P\ 2_1\ 2_1\ 2_1$	0.43
	14	0.2 M NaCl, 0.1 M Na Phos Cit 4.2 pH , 20 %w/v PEG 8000	1.5Å	69.51 78.52 82.73	$P\ 2_1\ 2_1\ 2_1$	0.24
	**15**	**0.17 M $(NH_4)_2SO_4$,15 %v/v Glycerol , 25.5 %w/v PEG 4000**	**1.5 Å**	**69.91 79.83 82.58**	$P\ 2_1\ 2_1\ 2_1$	**reference**

Table S2 Binding affinities of Fabs 12E1 and 10C3 towards NHBA variants

Kinetic parameters of Fabs 12E1 and 10C3 towards NHBA peptides 2, 3 and 20 calculated by SPR experiments are shown. SPR data were analysed using the Biacore T200 Evaluation software (GE Healthcare) and the U-values for all the measurements were below the value of 15, therefore indicating uniqueness in the K_D determination.

**Fab**	**Protein**	**Kon (1/M*s)**	**Koff (1/s)**	**U-value**	**K_D (M)**
12E1	NHBA p2	$6.1\ x10^{+4}$	$2.0x10^{-5}$	15	$3.3±0.8\ x10^{-10}$
	NHBA p3	$8.4x10^{+4}$	$3.1\ x10^{-4}$	5	$5.1±0.17x10^{-10}$
	NHBA p20	$4.9\ x10^{+4}$	$3.0\ x10^{-4}$	1	$6.2±1.5\ x10^{-9}$
10C3	NHBA p2	$2.37\ x10^{+5}$	$9.2\ x10^{-4}$	2	$5.5\ ±0.5x10^{-9}$
	NHBA p3	$4.34\ x10^{+5}$	$5.41x10^{-4}$	1	$1.2±0.16x10^{-10}$
	NHBA p20	No binding			

Table S3 CDRs loop conformation of Fabs 12E1 and 10C3

The CDRs of Fab 12E1 (*a*) and Fab 10C3 (*b*) are shown as identified and classified according to the cluster definition introduced by (North *et al.*, 2011). Loop conformation is assigned on the basis of the region of the Ramachandran plot for every CDR residue. B stands for β-sheet region, P for polyproline II, A for α-helix, D for δ region (near α-helix but at more negative values of ϕ), L for left-handed helix, and G for γ region (ϕ>0° excluding the L and B regions). *Cis* conformation is indicated by lower case letters.

(*a*)

Fab 12E1

CDR loop	Residues	Cluster	Loop Conformation
CDR-H1	Lys23–His35	H1-13-1	PPBLBPAAABPBB
CDR-H2	Trp50–Lys59	H2-10-1	BBPAADLPBB
CDR-H3	Ile97-Pro104	Not assigned	BPAGLLBp
CDR-L1	Arg24–Asn39	L1-16-1	BPBLPBPAPLLPPBBPB
CDR-L2	Tyr54–Ser61	L2-8-1	BLLDPPPP
CDR-L3	Met94–Thr102	L3-9-cis7-1	BBDABPpPB

(*b*)

Fab 10C3

CDR loop	Residues	Cluster	Loop Conformation
CDR-H1	Lys23-His35	H1-13-1	PPBLPAAABPBB
CDR-H2	Trp50-Asn59	H2-10-1	BBPDDDLPBB
CDR-H3	Ala96-Tyr103	H3-7-1	BPGADLAB
CDR-L1	Thr23-Ser36	L1-14-2	BBBAADAADBDPPB
CDR-L2	Tyr51-Ser58	L2-8-1	BLLDPPPP
CDR-L3	Ser91-Val100	L3-10-1	BBPDGLLPPB

Table S4 Amino-acidic composition of the CDRs of Fabs 12E1 and 10C3

The contribution of single amino acids or groups of amino acids to the total solvent accessible area (ASA) as calculated by PISA (Krissinel & Henrick, 2007) are expressed both in percentage and Å^2, and are clustered on the basis of their biochemical properties. The total ASA is calculated as the sum of the ASA of each residues belonging to the CDRs.

(*a*)

Fab 12E1

**Type**	**% on ASA**	**Amino acid**	**ASA (Å^2)**	**% on ASA**
		Tyr	373.35	9.70
Aromatic	18.93	Trp	240.42	6.25
		Phe	114.77	2.98
Positive		Lys	513.73	13.35
	28.84	His	144.41	3.75
		Arg	451.67	11.74
Negative	7.72	Asp	239.15	6.21
		Glu	57.85	1.50
		Ser	378.39	9.83
		Asn	290.85	7.56
Polar uncharged	28.97	Gln	103.75	2.70
		Thr	225.57	5.86
		Pro	116.60	3.03
		Cys	0.00	0.00
		Ile	262.38	6.82
		Gly	176.57	4.59
Non polar	15.54	Leu	88.66	2.30
		Val	36.67	0.95
		Ala	8.99	0.23
		Met	24.99	0.65
Total	100.00		3848.77	100.00

(*b*)

Fab 10C3

**Type**	**% on ASA**	**Amino acid**	**ASA (Å^2)**	**% on ASA**
Aromatic	18.16	Tyr	422.48	11.71
		Trp	218.67	6.06
		Phe	14.05	0.39
Positive	13.47	Lys	101.79	2.82
		His	134.04	3.72
		Arg	249.96	6.93
Negative	8.14	Asp	227.42	6.30
		Glu	66.07	1.83
Polar uncharged	46.53	Ser	718.75	19.92
		Asn	547.36	15.17
		Gln	0.00	0.00
		Thr	381.09	10.56
		Pro	31.46	0.87
		Cys	0.00	0.00
Non polar	13.71	Ile	51.88	1.44
		Gly	202.27	5.61
		Leu	51	1.41
		Val	185.44	5.14
		Ala	3.86	0.11
		Met	0.00	0.00
Total	100.00		3607.59	100.00

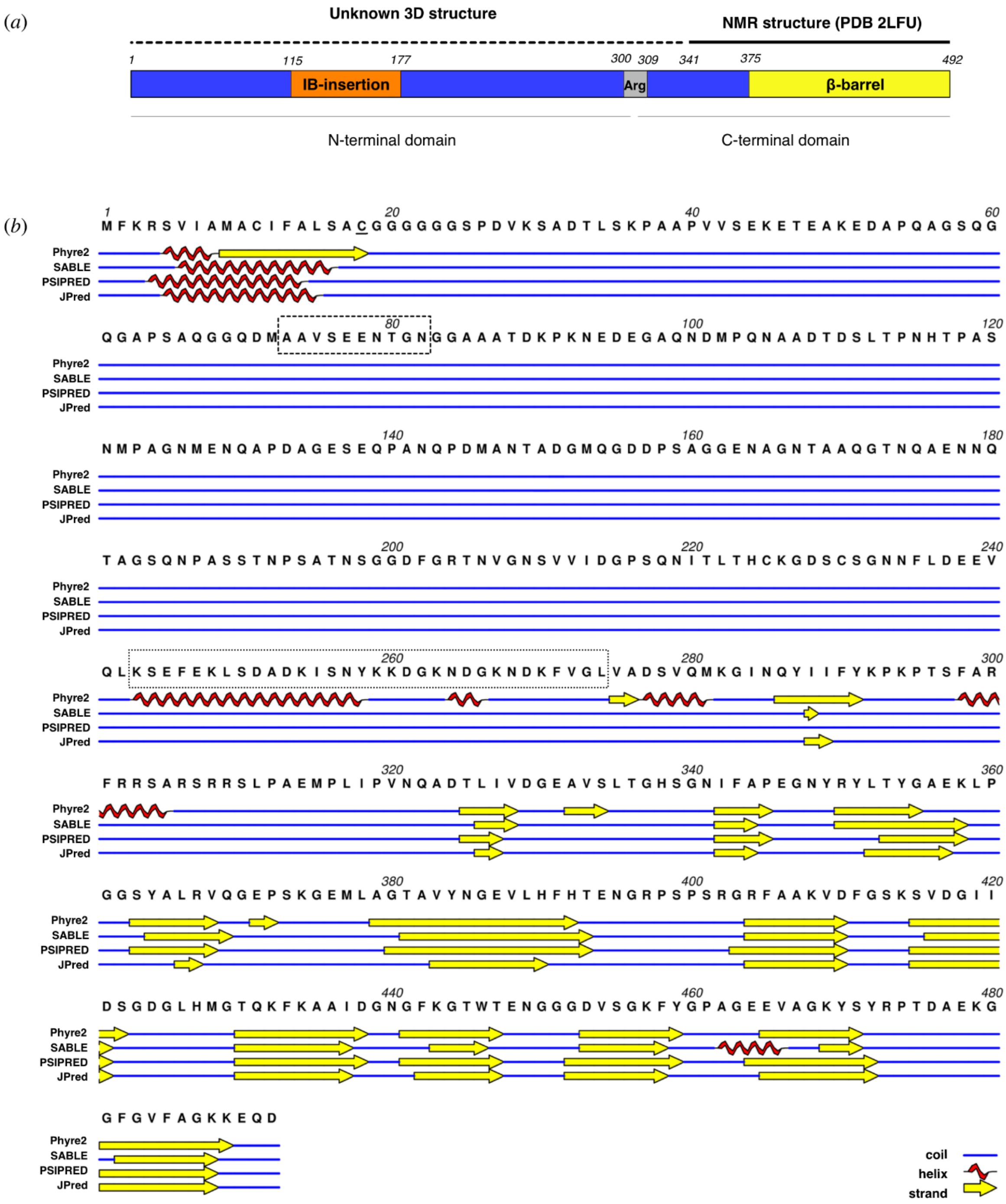

Figure S1 NHBAp2 and secondary structures predictions. (*a*) The NHBAp2 domain organization and structural coverage are depicted with a colored bar. Blue and yellow are for the N- and C-terminal domains, respectively, while orange and grey boxes show the location of the IB-insertion and the Arg-rich motif. (*b*) Secondary structure predictions on NHBAp2 sequence were performed using: Phyre2 (Kelley *et al.*, 2015), SABLE (Adamczak *et al.*, 2005), PSIPRED (Buchan *et al.*, 2013) and JPred (Drozdetskiy *et al.*, 2015). Putative epitopes of 12E1 and 10C3 mapped by Giuliani et al. (Giuliani *et al.*, in preparation) are boxed with a dashed line and a dotted line respectively. Cys18 (underlined) is

the NHBA lipidation site and residues preceding such Cys are cleaved in the mature surface-exposed form of the protein.

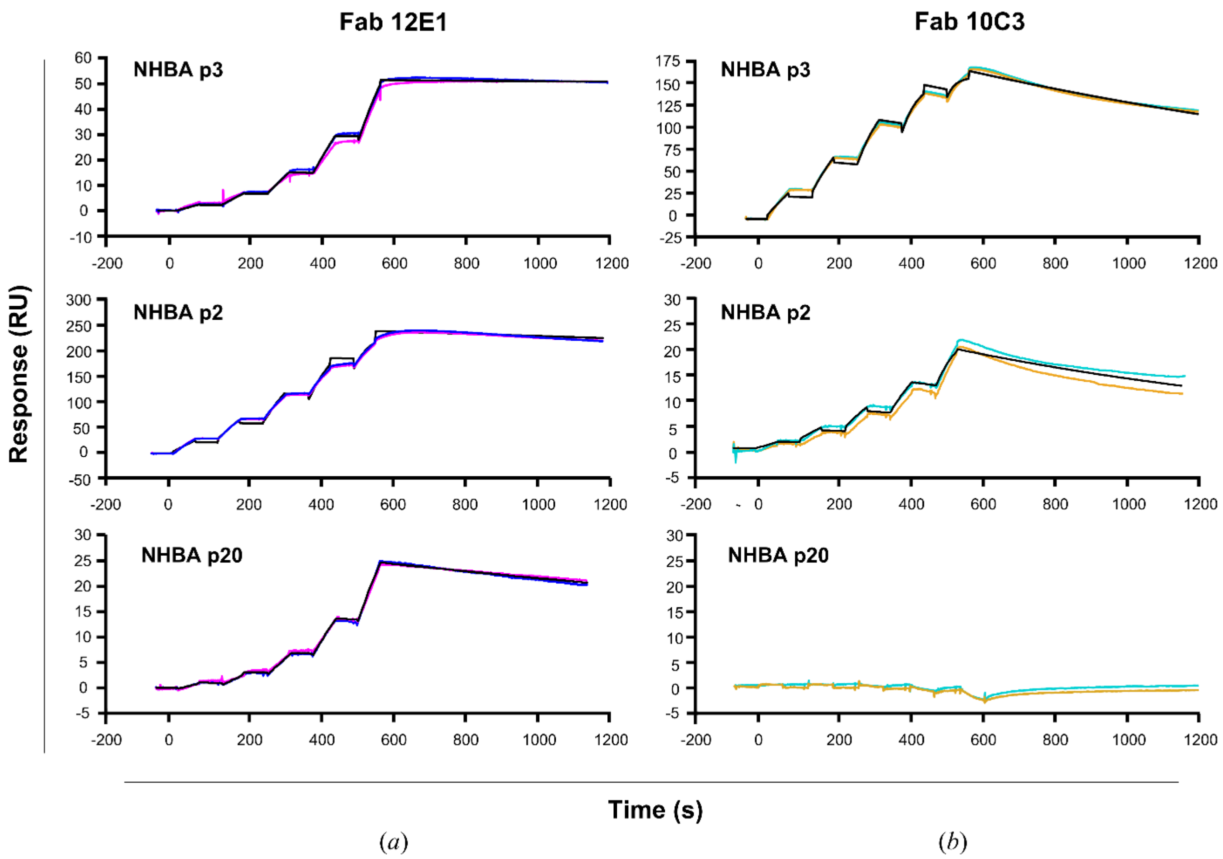

Figure S2 SPR sensorgrams of Fab10C3 and Fab12E1 and NHBA peptides 2, 3, and 20. Surface plasmon resonance (SPR) was used to determine the dissociation constants (K_D), using the single cycle kinetic (SCK) approach, for the NHBA variants p2, p3 and p20. The titrations included NHBA concentrations from 6.25 to 100 nM. Sensograms referring to Fab12E1 and 10C3 are plotted in the first (*a*) and the second (*b*) column respectively. Colored curves represent the experimental data, black lines represent the fitted curves. Note that the K_D of Fab10C3 towards NHBAp20 could not be measured due to lack of recognition of this variant.
